# Intracerebral hemorrhage CT scan image segmentation with HarDNet based transformer

**DOI:** 10.1038/s41598-023-33775-y

**Published:** 2023-05-03

**Authors:** Zhegao Piao, Yeong Hyeon Gu, Hailin Jin, Seong Joon Yoo

**Affiliations:** Department of Computer Science and Engineering, Sejong University, Seoul, South Korea

**Keywords:** Diseases, Computer science, Machine learning, Classification and taxonomy

## Abstract

Although previous studies conducted on the segmentation of hemorrhage images were based on the U-Net model, which comprises an encoder-decoder architecture, these models exhibit low parameter passing efficiency between the encoder and decoder, large model size, and slow speed. Therefore, to overcome these drawbacks, this study proposes TransHarDNet, an image segmentation model for the diagnosis of intracerebral hemorrhage in CT scan images of the brain. In this model, the HarDNet block is applied to the U-Net architecture, and the encoder and decoder are connected using a transformer block. As a result, the network complexity was reduced and the inference speed improved while maintaining the high performance compared to conventional models. Furthermore, the superiority of the proposed model was verified by using 82,636 CT scan images showing five different types of hemorrhages to train and test the model. Experimental results showed that the proposed model exhibited a Dice coefficient and IoU of 0.712 and 0.597, respectively, in a test set comprising 1200 images of hemorrhage, indicating better performance compared to typical segmentation models such as U-Net, U-Net++, SegNet, PSPNet, and HarDNet. Moreover, the inference time was 30.78 frames per second (FPS), which was faster than all en-coder-decoder-based models except HarDNet.

## Introduction

Intracerebral hemorrhage (ICH) is the condition caused by bleeding in the ventricles of the brain when blood vessels rupture spontaneously due to reasons other than external injury. ICH occurs primarily in middle-aged adults and is the sub stay of stroke, exhibiting the second highest occurrence rate after ischemic stroke^1^ owing to the high incidence, mortality, and disability rates. ICH can be categorized into five types based on the bleeding location within the brain: epidural hemorrhage (EDH), subdural hemorrhage (SDH), subarachnoid hemorrhage (SAH), intraventricular hemorrhage (IVH), and intraparenchymal hemorrhage (IPH). Given that ICH has become a life threatening disease and causes a burden on the families of those suffering from the disease, it is essential to develop accurate and rapid diagnosis and treatment methods for ICH.

A computed tomography (CT) scan is a fast diagnostic imaging technique having good resolution used for accurately determining the location of hematoma, amount of bleeding, the mass effect, presence or absence of bleeding in the ventricles, and the amount of damage to the subarachnoid and surrounding brain tissues. Therefore, it is considered ideal for the diagnosis and treatment of ICH^2^. Generally, experts first confirm the presence of hemorrhage through CT scans followed by detecting the type and location of the bleeding. However, a diagnosis as such requires extensive time from a radiology specialist for the examination, especially when it entails the possibility of a missed diagnosis.

Medical image segmentation is the process of identifying areas affected by the disease using medical diagnosis technologies such as computed tomography (CT) or magnetic resonance imaging (MRI). While existing deep learning-based ICH image segmentation (hereinafter referred to as “ICH segmentation”) methods using the U-shaped encoder-decoder architecture acquired adequate results, two problems still persisted. First, these networks require much time for inference and training owing to a large number of parameters. The inference time increases for high resolution input images. Second, when low-resolution features extracted from the encoder are transformed into high-resolution features in the decoder, it results in the significant loss of sensitivity to the sensitivity of the final segmentation.

Because ICH must be definitively diagnosed and treated within 1*h* of its occurrence, the speed of the diagnosis model is critical when diagnosing ICH, in addition to the performance. Therefore, this study proposes a TransHarDNet ICH segmentation network to overcome such drawbacks for the effective diagnosis and treatment of ICH. TransHarDNet comprises a U-shaped encoder-decoder architecture and has the following characteristics: The existing convolution calculation is replaced with a transformer block with a self-attention mechanism for the effective exchange of information between the encoder and the decoder^3^. Long-distance dependency can be modeled, and global information is analyzed to extract various context features and produce more detailed segmentation results.

In this study, we used 82,636 CT scan images of ICH as datasets from five different institutions, including the Catholic University of Korea Seoul St. Mary’s Hospital. Furthermore, we compared the inference speed and segmentation performance of the TransHarDNet model with that of other segmentation models, such as the U-Net^4^, U-Net++^5^, SegNet^6^, PSPNet^7^, and HarDNet^8^. Experimental results showed that the TransHarDNet model exhibited an inference speed of 30.78 FPS, IoU of 0.597, and a Dice coefficient of 0.712, which makes it superior to other conventional models.

## Related works

In an ICH image analysis, segmentation accurately detects the bleeding location amount in the initial step of identifying the occurrence of bleeding, which is why it has more clinical applicability than classification. Segmentation techniques are also used to analyze medical diagnostic images except that of ICH. The most frequently used segmentation models include those having an encoder-decoder architecture that has been transformed based on U-Net.

U-Net^4^, a segmentation model proposed in 2015, uses a symmetric encoder-decoder architecture with a skip connection, and exhibits outstanding performance in the seg-mentation of medical images by converging multiscale features. Other U-Net shaped models based on U-Net, such as U-Net++^5^, 3D U-Net^9^, and Attention U-Net^10^, have been widely used owing to their excellent performance in the analysis of medical images. Furthermore, models such as SegNet^9^ and PSPNet^10^ having an encoder-decoder architecture have also been widely adopted. Zhang et al.^11^ proposed a technology that used a generator net to generate an ICH image, which was further synthesized along with a normal ICH image having an insufficient amount of training data using the U-Net-based network. Results showed that the performance could be improved if the ICH detection model was trained on the synthesized and actual data simultaneously. This however was a new case wherein U-Net was applied to a medical image synthesis in addition to segmentation. Kushnure and Talbar^12^ conducted a study and accurately extracted the global and local feature information from CT scan images by replacing the CNN block of the U-Net and combining Res2Net^13^ and a squeeze-and-excitation (SE) network. Abramova et al.^14^ proposed a segmentation model using 3D U-Net, where the SE network was applied to U-Net. You et al.^15^ proposed a 3D Dissimilar-Siamese-U-Net comprising two U-Nets connected to the encoder by a distance block. The brain CT scan images were analyzed in the 3D Dissimilar-Siamese-U-Net by receiving two inputs: left and right. Mizusawa et al.^16^ conducted a study wherein U-Net was applied for the reconstruction of an X-ray image.

Recurrent neural networks (RNN), a model architecture for processing sequence data, have been widely used in natural language processing (NLP). Because CT scan or MRI images are established as continuous slices in medical image analyses they can be analyzed using RNNs. Stollenga et al.^17^ conducted a study for the segmentation of brain MRI images using the 3D PyraMiDLSTM model. The network was constructed to enable GPU-based parallel processing to significantly improve the efficiency of model training, which produced good segmentation results in the MRBrainS challenge^18^. Koutnìk et al.^19^ constructed a spatial clockwork recurrent neural network (CW-RNN) using fewer parameters than RNNs for the segmentation of muscular disease images. As a result, the average accuracy of CW-RNN was 5% higher than that of U-Net, and the execution speed was 100 times shorter than that of the CNN models. Poudel et al.^20^ constructed recurrent fully convolutional networks based on FCN and RNNs for the real-time computing of heart segmentation.

Chen et al.^21^ performed CT image segmentation using TransUNet, developed by combining U-Net with 12 transformer layers and obtained outstanding results. Wang et al.^22^ built a 3D MRI brain tumor segmentation model with a transformer architecture based on U-Net. Chen et al.^21^ and Wang et al.^22^ proved that the overall performance of the segmentation model can be improved by combining a CNN model having an encoder-decoder architecture with a transformer used for the analysis of sequence data. However, Wang et al.^22^ used a 3D CNN layer with a large number of parameters and exhibited a slow processing time considering the existing U-Net model architecture was applied.

### Dataset

**Figure 1:**
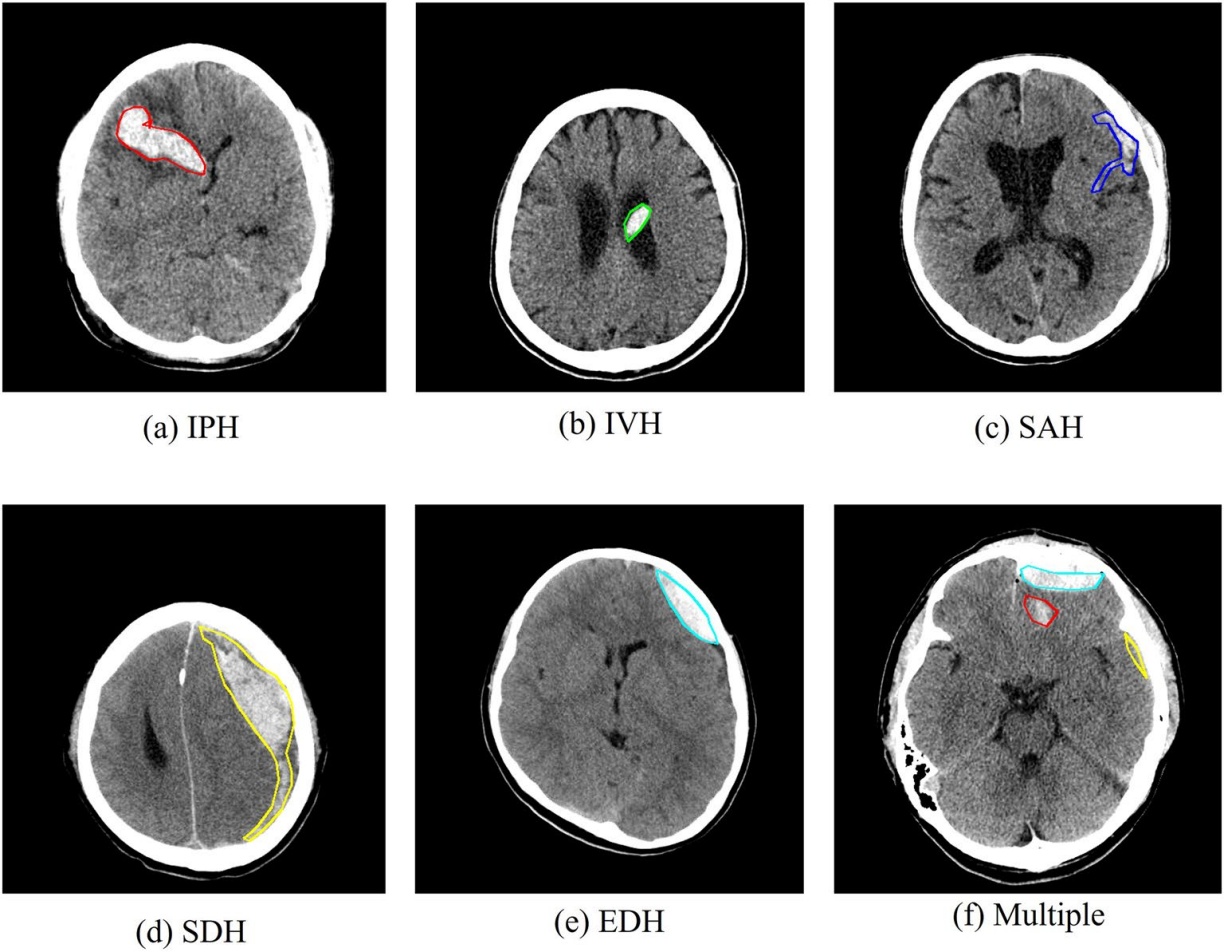
Data examples; (**a**) example of intraparenchymal hemorrhage (IPH); (**b**) example of intraventricular hemorrhage (IVH); (**c**) example of subarachnoid hemorrhage (SAH); (**d**) example of subdural hemorrhage (SDH); (**e**) example of epidural hemorrhage (EDH); (**f**) CT images with one or more cerebral hemorrhagic lesions (the image include IPH, SDH, and EDH).

In this study, we used 82,636 CT scan images of ICH as datasets, collected from the Catholic University of Korea Seoul St. Mary’s Hospital, Chung-Ang University, Inje University, Inje University Pusan Paik Hospital, and Konkuk University Medical Center(The dataset published on AIHub^23^). For the data, experts manually found the disease area and marked the ground truth. All images were high-resolution (512 ± 512) and were categorized as EDH, IPH, IVH, SAH, or SDH depending on the location of bleeding. Figure 1a–f show examples of the CT scan images from each category, that is, intraparenchymal hemorrhage (IPH), intraventricular hemorrhage (IVH), subarachnoid hemorrhage (SAH), subdural hemorrhage (SDH), epidural hemorrhage (EDH), and at least one type of hemorrhage (multiple), respectively.

The 200 ICH images were selected from the EDH, IPH, IVH, SAH, SDH, and multiple categories to acquire 1200 images for the test data. The remaining 81,436 images were divided in a ratio of 8:2 at the unit of disease category to form training and validation datasets comprising 65,151 and 16,285 images, respectively. Table 1 shows the statistics of the data used for the training and testing of a model in each category.

**Table 1:** Our ICH dataset.

	EDH	IVH	SDH	SAH	IPH	Multiple	Sum
Train	2286	5352	27,413	13,421	17,605	15,359	81,436
Test	200	200	200	200	200	200	1200
Sum	2486	5552	27,613	13,621	17,805	15,559	82,636

## The proposed model: TransHarDNet

This study proposed the TransHarDNet segmentation model to accurately and quickly generate segmentation results for the CT scan images of ICH. Figure 5 shows a schematic of TransHarDNet. The model comprises an encoder-decoder-based U-Net architecture. HarDNet was used as the backbone of the encoder-decoder owing to its light-weight architecture. The simple convolution calculation was replaced with a transformer block that connected the encoder and the decoder. Table 2 is the details of the model architecture.

**Table 2:** Model architecture.

Stage	Block name	Details	Output size
Input	–	–	512 × 512 × 1
Encoder	Conv block	4 × convolution	128 × 128 × 48
	HarDNet block	4 × convolution	128 × 128 × 48
	Down sampling block	Convolution, AvgPool2d	64 × 64 × 64
	HarDNet block	4 × convolution	64 × 64 × 78
	Down sampling block	Convolution, AvgPool2d	32 × 32 × 96
	HarDNet block	8 × convolution	32 × 32 × 160
	Down sampling block	Convolution, AvgPool2d	16 × 16 × 160
	HarDNet block	8 × convolution	16 × 16 × 214
	Down sampling block	Convolution, AvgPool2d	8 × 8 × 224
	HarDNet block	8 × convolution	8 × 8 × 286
Transformer (bottle neck)	–	1 × convolution	8 × 8 × 320
	Linear projection	Reshape	512 × 64
	Transformer block	4 × transformer layer	512 × 64
	–	1 × convolution	8 × 8 × 512
	–	1 × convolution	8 × 8 × 320
Decoder	Up sampling block	Upsample, convolution	16 × 16 × 320
	HarDNet block	8 × convolution	16 × 16 × 214
	Up sampling block	Upsample, convolution	32 × 32 × 214
	HarDNet block	8 × convolution	32 × 32 × 160
	Up sampling block	Upsample, convolution	64 × 64 × 160
	HarDNet block	4 × convolution	64 × 64 × 78
	Up sampling block	Upsample, convolution	128 × 128 × 78
	HarDNet block	4 × convolution	128 × 128 × 48
	Up sampling block	Upsample, convolution	512 × 512 × 6
Output	Conv block	1 × convolution	512 × 512 × 6

### HarDNet block

HarDNet is a densely connected network architecture built to maintain high accuracy while reducing memory usage. Compared to methods such as DenseNet block or ResNet block, HarDNet block can shorten the inference time by approximately 30% at a similar performance level in applications such as image classification, object detection, and image segmentation^8^.

**Figure 2:**
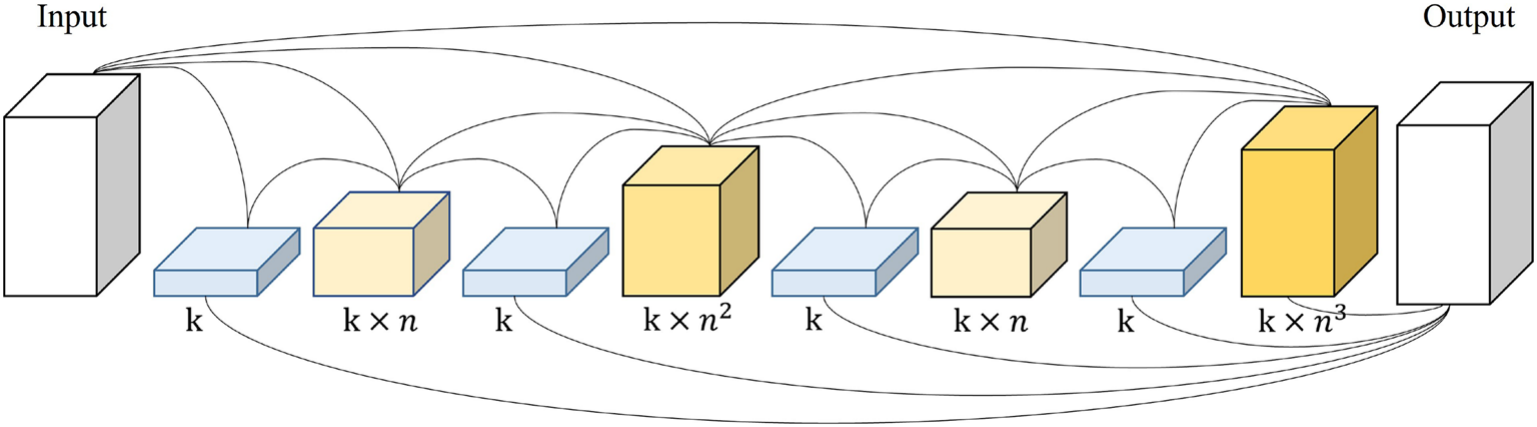
Example of HarDNet connections.

HarDNet comprises harmonic dense blocks (HDBs), which are connected when the *k*-th layer is connected to the $k-2^n$-th layer, when $k-2^n$ is greater than 0 and $\frac{2^n}{k}$ is a natural number, as seen in (1). *k* is the location of a layer in the HDB, *n* is the layer connected to k in the HDB, and *N* is a natural number. And Fig. 2 is an illustration of HarDNet.

1 $$\begin{aligned} {C_k=k-2^n}, if {2^n \over k} \in N, {k-2^n \ge 0} \end{aligned}$$

**Figure 3:**
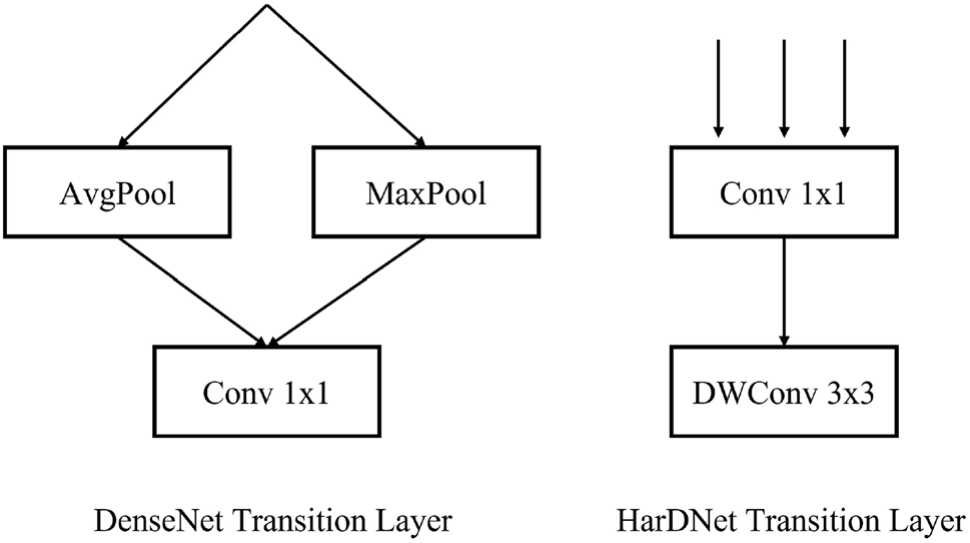
Comparison of the transition layers of DenseNet and HarDNet.

In HarDNet, HDBs are connected by a depth wise-separable convolution layer (DWConv), which reduces the convolutional input/output (CIO) by 50% when compared to the $1 \times 1$ convolution layer. Therefore, a $2 \times 2$ average pooling layer is used in DenseNet[3]. Figure 3 is a comparison of the transition layers of DenseNet and HarDNet.

### Transformer block

**Figure 4:**
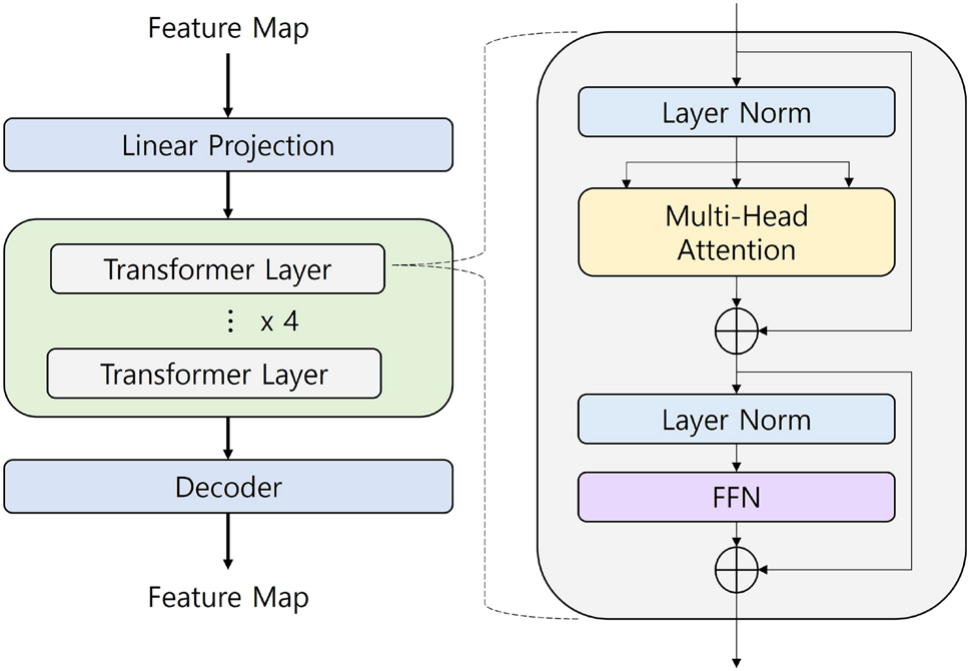
Architecture of transformer.

In the transformer, the sequence data processing method used in NLP analysis was successfully applied to computer vision. Currently, Owing to their outstanding performance, transformers are gaining wide attention in computer vision for applications such as detection^24^, segmentation^25^, and classification^26^. In this study, the existing CNN connection between the U-NET encoder and decoder was replaced with a segmentation transformer (SETR). The SETR architecture is shown in Fig. 4.

A feature map extracted from the encoder using HarDNet as a backbone was input to the transformer. The feature map is transformed into sequence data in the linear projection block using the position-embedding technique. The sequence data comprising information on the location were first normalized through layer normalization (LN), which resolved the internal covariate shift (ICS) occurring during the training of small batches. Furthermore, the normalized data is input to a multi-head attention block to extract various features by inferring the relationship between the location information in the sequence data.

The output of the multi-head attention block and the first sequence data delivered through the skip connection are combined and passed through the LN and feed-forward network (FFN). The FFN comprises two activation functions: the first layer is the ReLU activation function, and the second layer is a linear activation function that facilitates inference. One transformer layer was configured as such. In this study, we constructed a module with four transformer layers, and the feature map passing through these layers is decoded to the same size as the input.

### Model architecture

TransHarDNet comprises an encoder, a decoder, and a bottleneck layer.

The encoder extracts the feature map and reduces the image size through down sampling. The encoder comprises a convolution block and the HarDNet block. W, H, and C represent the width, height, and channel of the preprocessed image (W, H, C). The shape of the feature map inferred with a convolution block was W/4, H/4, C*48. The convolution block consists of a convolution layer where filter = 16, kernel size = 3, stride = 2, a convolution layer where filter = 24, kernel size = 3, stride = 1, a convolution layer where filter = 32, kernel size = 3, stride = 2, and a convolution layer where filter = 48, kernel size = 3, and stride = 1. A down-sampling block consists of a convolution layer with kernel size = 1 and an AvgPoll2d layer with kernel size = 2 and stride = 2. Subsequently, the feature map undergoes down sampling through the HarDNet block and results in W/32, H/32, C*320.

The transformation section extracts valid information from the feature map and delivers it to the decoder. The feature map is encoded into sequence data through a linear projection layer and passed through four transformer layers. The sequence data is decoded by two convolution layers where kernel size = 1, stride = 1 again to the dimensions of W/32, H/32, C*320 and delivered to the TransHarDNet decoder.

**Figure 5:**
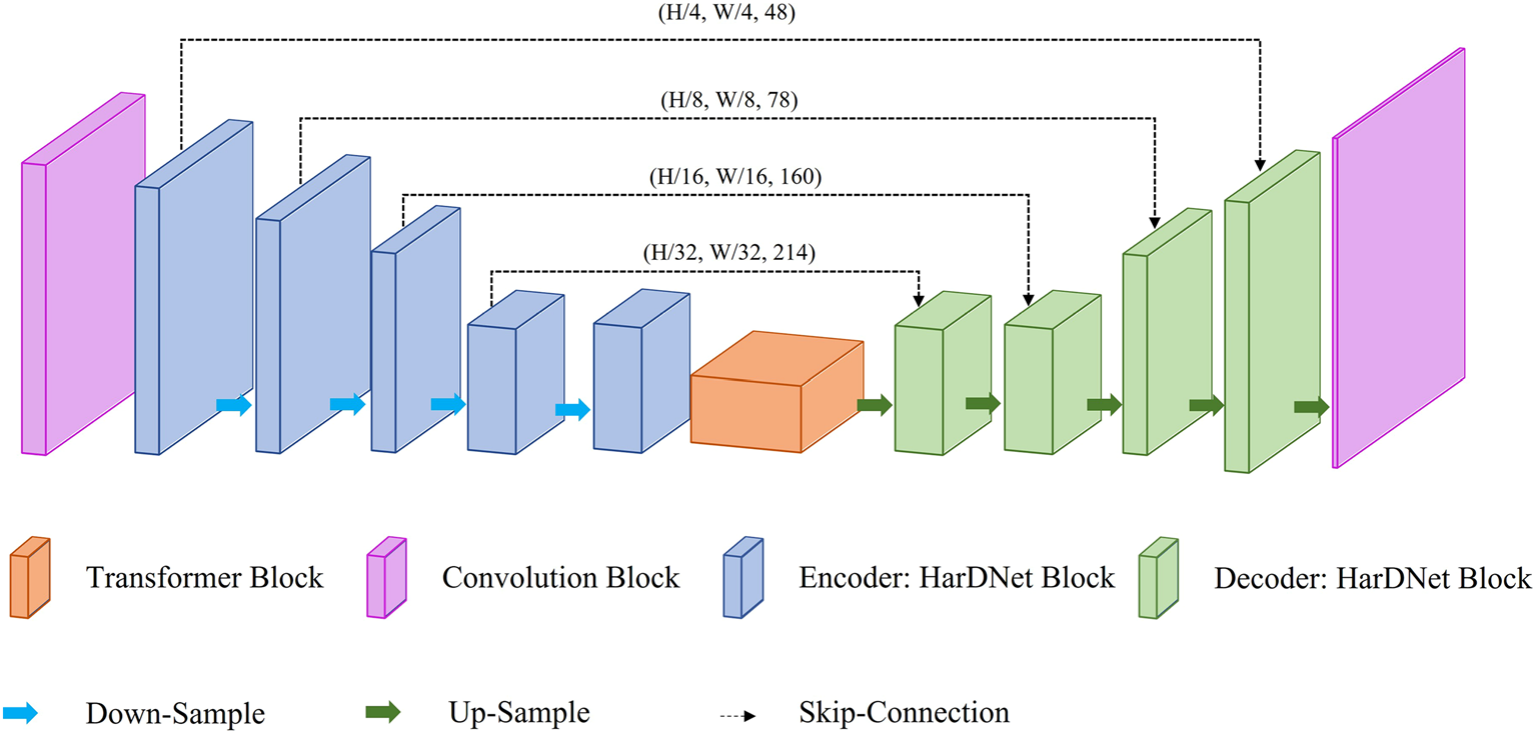
Overall architecture of the proposed TransHarDNet.

The feature map from the transformer block is up-sampled by the decoder to the same size as the TransHarDNet input, while the ICH region of the feature map is marked in the output image. The decoder outputs the final (W, H) image size by passing through four HarDNet blocks, five up-sampling blocks, and the last convolution layer with kernel size = 1. An up-sampling block consists of an interpolate function that uses the “bilinear” mode and a convolution layer with kernel size = 1. Figure 5 and Table 2 are detailed descriptions of the HarDNet structure.

## Experimentations

### Performance evaluation indicators

We evaluated the performance of the model based on four indicators commonly used to evaluate the performance of a model in medical image segmentation: the Dice similarity coefficient (DSC), intersection over union (IoU), Jaccard index, precision, and recall. IoU is calculated as the ratio of the intersection value of the predicted and actual values to the union value and is used for object detection and semantic segmentation. The Dice coefficient, IoU, precision, and recall can be inferred using true positive (TP), true negative (TN), false positive (FP), and false negative (FN) indicators of a confusion matrix. The Dice coefficient, IoU, precision, and recall can be calculated as shown in (2)–(5).

2 $$\begin{aligned} Dice= & {} {2TP \over (2TP+FP+FN) } \end{aligned}$$

3 $$\begin{aligned} IoU=  {TP \over (TP+FP+FN)} \end{aligned}$$

4 $$\begin{aligned} Precision=  {TP\over (TP+FP)} \end{aligned}$$

5 $$\begin{aligned} Recall= {TP\over (TP+FN)} \end{aligned}$$

### Experimental environment and parameter setting

Table 3 presents the experimental environment used for this study. The input image size for all models was 515 ± 512 for training, and the batch size was 8. Adaptive moment estimation (Adam) was used as the optimization algorithm for training the model, and the initial learning rate was set to 0.01. The learning rate was reduced by 0.5 when the training loss value did not decrease for five epochs, and early stopping was applied when the value did not decrease for 10 epochs.

**Table 3:** Experimental environment.

Device	Specifications
OS	Windows 10
CPU	Intel Core i9-9900KF 3.6 GHz
GPU	NVIDIA GeForce RTX 2080Ti * 1
RAM (memory)	96 GB
Storage	1TB SSD + 4TB HDD
Language	Python 3.7, PyTorch = 1.5

### Selection of a loss function

We conducted an experiment to determine an appropriate loss function for the model by combining the Dice loss, cross entropy (CE), and focal loss^27^.

The Dice loss, which stems from the Dice coefficient, was first proposed in a study by Milletari et al.^28^ and is widely used in medical image segmentation. In this study, the Dice loss was used to indicate similarities between the two samples. The Dice loss value ranges between 0 and 1, and a smaller value indicates a higher level of similarity between the two samples. It can be calculated using (6):

6 $$\begin{aligned} L_{Dice} (X,Y) \;=\; 1\; - \; {{2 \; | X \bigcap Y |} \; \over \; {| X | \; + \; | Y |}} \end{aligned}$$

The concept of CE originated from information theory, an expanded concept of binary cross entropy frequently used in multinomial classification. As seen from (7), the CE loss function infers the difference in the quantity of information between the predicted and actual values of the sample, where M is the number of categories, $y_{ic}$ is the dummy variable having a value of 1 with identical predicted and actual values, and 0 otherwise, and pic is the probability of category *c* for input *i*.

7 $$\begin{aligned} L_{CE} \;=\; {1 \over N} \sum _{c=1}^m y_{ic} log(p_{ic}) \end{aligned}$$

Focal loss is a loss function first used for object detection, and since, has been used to solve category imbalance issues and differences in category difficulty of classification problems. As shown in (8), the focal loss adds a modulating factor based on weight cross entropy (WCE) to reduce the weight of samples that can be classified easily during training to focus on the samples difficult to classify.

8 $$\begin{aligned} L_F \;=\; - \; (1-p_t)^r log(p_t) \end{aligned}$$

The losses in (9) and (10), referred to as DiceCE and DiceFocal, respectively, were combined to perform the experiment in this study. Two loss functions were applied to the model for training, and the performance was measured using the test dataset. The results are provided in Table 4. Compared to DiceFocal, the model with the DiceCE loss function applied produced improved results for all four performance indicators. Therefore, the DiceCE loss function was used in this study.

9 $$\begin{aligned} L_1= & {} L_{Dice} + L_{CE} \end{aligned}$$

10 $$\begin{aligned} L_2= & {} L_{Dice} + L_{Focal} \end{aligned}$$

Two loss functions were applied to TransHarDNet for training, and the performance of the model was measured using the test dataset. The results are provided in Table 4. With an average Dice coefficient of 0.712, IoU of 0.597, precision of 0.777, and recall of 0.708, TransHarDNet exhibited better performance when DiceCE was applied compared to when DiceFocal was applied. Furthermore, DiceCE produced better results for each ICH category compared to DiceFocal. Therefore, the DiceCE loss function was used in TransHarDNet for the following experiments.

**Table 4:** Comparison of performance by category of the proposed models.

	Dice		IoU		Precision		Recall	
	DiceCE	DiceFocal	DiceCE	DiceFocal	DiceCE	DiceFocal	DiceCE	DiceFocal
EDH	0.777	0.709	0.681	0.614	0.809	0.786	0.772	0.684
IPH	0.809	0.770	0.714	0.676	0.845	0.832	0.821	0.752
IVH	0.742	0.675	0.625	0.566	0.810	0.761	0.734	0.656
SAH	0.545	0.471	0.414	0.353	0.643	0.615	0.554	0.454
SDH	0.709	0.618	0.591	0.505	0.766	0.742	0.712	0.586
Multicategory	0.686	0.657	0.557	0.528	0.783	0.785	0.653	0.609
Average	0.712	0.650	0.597	0.540	0.777	0.754	0.708	0.623

### Comparative analysis for the model performance

We conducted a comparative analysis by measuring the segmentation performance for the TransHarDNet model proposed in this study and the conventional segmentation models such as U-Net^4^, U-Net++^5^, SegNet^18^, PSPNet^19^, and HarDNet^8^ to verify the effectiveness of the proposed model. Furthermore, we used identical hyper-parameters and DiceCE loss function to ensure consistency in the training process.

The experimental results are listed in Table 5. The proposed TransHarDNet exhibited better performance than the four conventional semantic methods, with Dice coefficients, IoU, and HD95 of 0.712, 0.597, and 27.733, respectively. Furthermore, the TransHarDNet, wherein a transformer module was introduced to HarDNet, improved the model accuracy by 1.6% compared to HarDNet alone by applying simple convolution calculation.

**Table 5:** Comparative analysis for the models.

Model	Dice	IoU	HD95
U-Net	0.684	0.569	30.693
U-Net++	0.676	0.561	32.005
SegNet	0.588	0.480	33.391
PSPNet	0.709	0.593	27.886
HarDNet	0.708	0.591	28.609
SwinTransformer	0.710	0.593	28.614
TransUNet	0.651	0.532	38.253
TransHarDNet (our)	0.712	0.597	27.733

**Figure 6:**
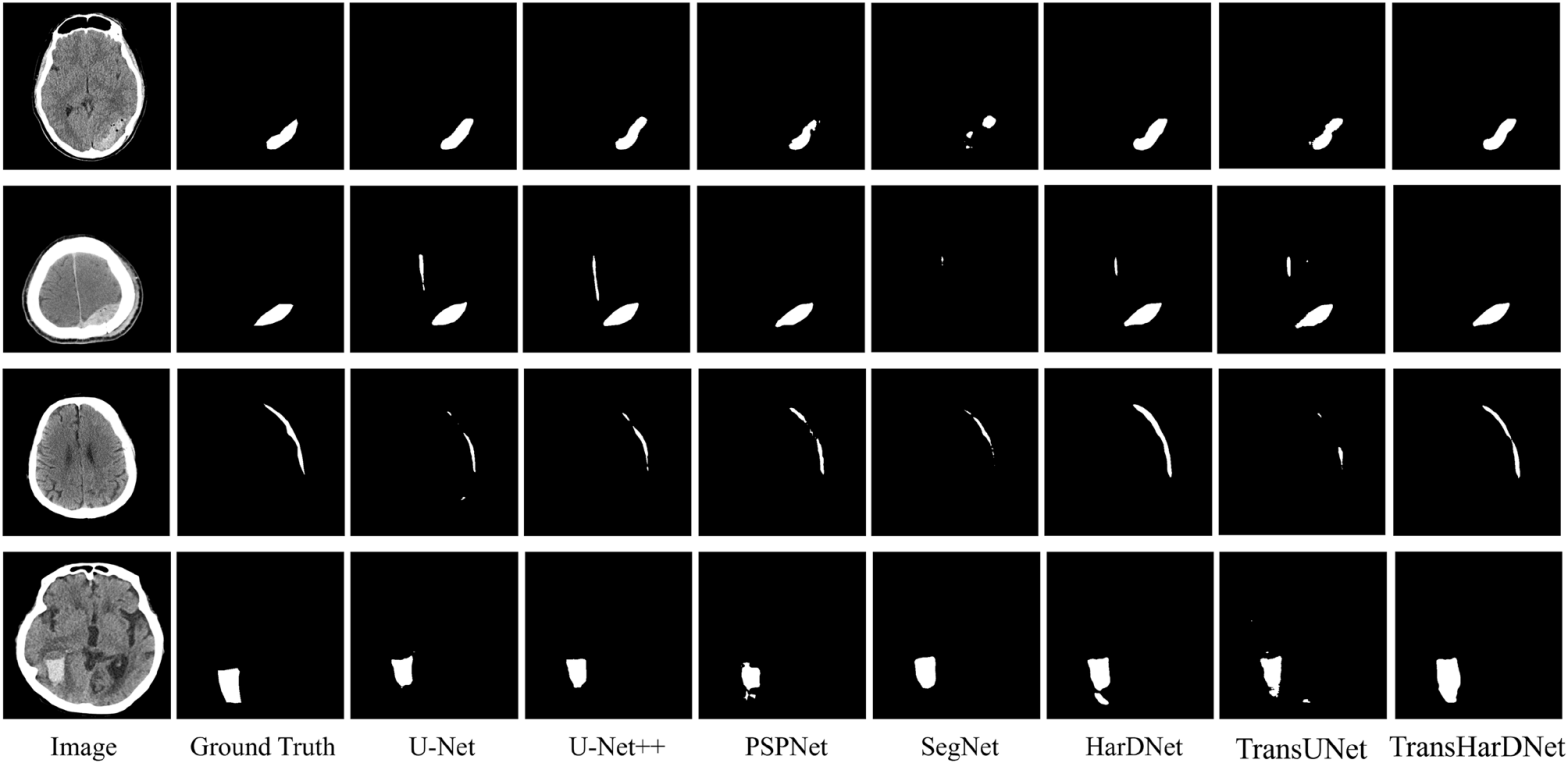
Example of segmentation results.

Figure 6 shows the prediction results, which intuitively represent the results of the semantic segmentation methods used in the experiment. The results showed that the TransHarDNet can segment the bleeding location more accurately compared to other segmentation methods.

### Comparative analysis for the model speed

Owing to a large number of parameters, existing segmentation models have limitations in terms of the complicated model architecture and slow inference speed. Additionally, while most segmentation analysis models require a 3-channel RGB image as input, the brain CT scan images are grayscale and exhibit a simple image type. Therefore, using models with complicated architecture and a large number of parameters to acquire brain CT scan images could reduce model efficiency and result in overfitting.

The model proposed in this study possessed the characteristics of HarDNet, which enables fast and accurate ICH segmentation. The total inference time, FPS, and the number of parameters for each semantic segmentation network were identified using the test dataset comprising 1200 images. As shown in Table 6, the inference time of TransHarDNet is 30.78, which is faster than most encoder-decoder-based segmentation networks, except for HarDNet. Furthermore, the inference speed improved by 44.64% compared to PSPNet, which is the second most outstanding segmentation model in terms of performance. With respect to the model size, TransHarDNet is lighter compared to other semantic segmentation models, except for HarDNet.

**Table 6:** Comparative analysis of the models speed.

Model	Inference times (s)	FPS	Model size (MB)
U-Net	49.0	24.49/s	69.07
U-Net++	51.5	23.30/s	36.65
SegNet	46.9	25.58/s	117.78
PSPNet	56.4	21.28/s	186.83
HarDNet	38.6	31.09/s	16.47
SwinTransformer	158.8	7.56/s	19.53
TransUNet	58.2	20.62/s	207.21
TransHarDNet (our)	39.0	30.78/s	108.09

### Discussion

In this study, we focused on improving the performance of ICH segmentation by connecting the HarDNet block and the transformer block. Also, the proposed model showed good performance in many categories except SAH in ICH segmentation. When SAH and multi-class are excluded, the proposed model exhibits more desirable performance with Dice coefficient of 0.759, IoU of 0.653, precision of 0.808, and recall of 0.760. But in this study, the reason for the low Dice and IoU performance in SAH was not confirmed. This will be addressed in future research.

## Results

In this study, we proposed a TransHarDNet model with a U-Net-based encoder-decoder architecture for the segmentation of ICH regions in the CT scan images of the brain. The conventional CNN block between the encoder and decoder was replaced with the HarDNet backbone. Furthermore, the part between the encoder and decoder connected through CNN calculation was replaced by a transformer block. By combining the HarDNet and transformer blocks, the TransHarDNet network complexity was reduced, which improved the inference speed while maintaining the high performance of the model.

Through the self-attention mechanism of the transformer, the proposed model can effectively analyze and model the feature map by learning the context in high-level semantics, thereby overcoming the drawbacks of extensive calculation and insufficient understanding of the context existing in conventional methods.

We used 82,636 CT scan images of five different types of ICH provided by the Catholic University of Korea Seoul St. Mary’s Hospital to verify the proposed model. Compared to conventional segmentation models such as U-Net, U-Net++, SegNet, PSPNet, and HarDNet, the TransHarDNet exhibited the best performance in all performance evaluation indicators with a Dice coefficient, IoU, and HD95 of 0.712, 0.597, and 27.733, respectively.

Moreover, the TransHarDNet has fewer parameters and maintains a high speed when using a HarDNet block. The inference speed of TransHarDNet was calculated as 30.78 FPS, which was 25.68% faster than U-Net, and the performance improved by 3%. Although the inference speed was 1.0% slower than that of the conventional HarDNet, the segmentation performance improved by 2%. Based on the acquired results, the effectiveness of the proposed TransHarDNet model proposed has been sufficiently proven.

## Data Availability

The datasets analysed during the current study are available in the [AIHub^23^] repository, [https://aihub.or.kr/aidata/34101], or available from the corresponding author on reasonable request.
